# Future of Artificial Intelligence in Surgery: A Narrative Review

**DOI:** 10.7759/cureus.51631

**Published:** 2024-01-04

**Authors:** Aamir Amin, Swizel Ann Cardoso, Jenisha Suyambu, Hafiz Abdus Saboor, Rayner P Cardoso, Ali Husnain, Natasha Varghese Isaac, Haydee Backing, Dalia Mehmood, Maria Mehmood, Abdalkareem Nael Jameel Maslamani

**Affiliations:** 1 Cardiothoracic Surgery, Harefield Hospital, Guy's and St Thomas' NHS Foundation Trust, London, GBR; 2 Major Trauma Services, University Hospital Birmingham NHS Foundation Trust DC, Birmingham, GBR; 3 Medicine, University of Perpetual Help System Data - Jonelta Foundation School of Medicine, Las Piñas, PHL; 4 Internal Medicine, Mayo Hospital, Lahore, PAK; 5 Medicine and Surgery, All India Institute of Medical Sciences, Jodhpur, Jodhpur, IND; 6 Radiology, Northwestern University, Lahore, PAK; 7 Medicine and Surgery, St John's Medical College Hospital, Rajiv Gandhi University of Health Sciences, Bengaluru, IND; 8 Medicine, Universidad de San Martin de Porres, Lima, PER; 9 Community Medicine, Fatima Jinnah Medical University, Lahore, PAK; 10 Internal Medicine, Shalamar Medical and Dental College, Lahore, PAK; 11 General Practice, Cairo University, Cairo, EGY

**Keywords:** automated artificial intelligence (autoai), general thoracic surgery, general and vascular surgery, ortho surgery, black box, artificial intelligence in surgery, aritifical intelligence, surgery general

## Abstract

Artificial intelligence (AI) is the capability of a machine to execute cognitive processes that are typically considered to be functions of the human brain. It is the study of algorithms that enable machines to reason and perform mental tasks, including problem-solving, object and word recognition, and decision-making. Once considered science fiction, AI today is a fact and an increasingly prevalent subject in both academic and popular literature. It is expected to reshape medicine, benefiting both healthcare professionals and patients. Machine learning (ML) is a subset of AI that allows machines to learn and make predictions by recognizing patterns, thus empowering the medical team to deliver better care to patients through accurate diagnosis and treatment. ML is expanding its footprint in a variety of surgical specialties, including general surgery, ophthalmology, cardiothoracic surgery, and vascular surgery, to name a few. In recent years, we have seen AI make its way into the operating theatres. Though it has not yet been able to replace the surgeon, it has the potential to become a highly valuable surgical tool. Rest assured that the day is not far off when AI shall play a significant intraoperative role, a projection that is currently marred by safety concerns. This review aims to explore the present application of AI in various surgical disciplines and how it benefits both patients and physicians, as well as the current obstacles and limitations facing its seemingly unstoppable rise.

## Introduction and background

With rapid advancements in modern technology, artificial intelligence (AI) is being widely integrated into all aspects of human life - particularly healthcare. Continuous research is being carried out on the applicability of AI in the fields of medicine and surgery, and its potential benefits in screening and intervention - with the ultimate goal of evolving the quality of patient care and safety [1].

For instance, studies have shown that the application of AI algorithms in specialties such as ophthalmology has ensured increased accuracy in the screening and diagnosis of certain pathologies, such as cataracts, glaucoma, and keratoconus. Moreover, evaluating the risk of post-surgical complications and selecting individuals for operative procedures, such as laser-assisted in situ keratomileusis (LASIK) and small incision lenticular extraction (SMILE), has been achieved, with an accuracy of 93% using AI and its subsets [2]. Research in orthopedics and neurosurgery has shown the importance of AI in the diagnostic and prognostic improvement of surgical procedures, such as total joint replacement and the management of brain tumors, respectively [3,4]. In a study, Kunze et al. [5] found an improvement in the accuracy of diagnostic performance in the detection of anterior cruciate ligament (ACL) and meniscus tears with the use of AI. The design of clinical trials and data mining have also been facilitated by AI techniques [6,7].

Machine learning (ML), a subset of AI, has gained tremendous popularity in healthcare, especially in the field of surgery. ML involves the use of algorithms and input data that can produce expected outputs. A study conducted in 2021 by Acumen Research and Consulting, on the global AI in the healthcare market, reported that a compound annual growth rate (CAGR) of 43.4% is expected during the period from 2022 to 2030, with the market size growing from 2021 value of USD 7.9 billion and reaching an estimated value of USD 201.3 billion by 2030 [8].

In this review, we aim to provide a comprehensive understanding of AI and ML in surgery to highlight their roles and uses in various surgical interventions, their effectiveness and limitations, and the potential enhancement of patient management and safety.

## Review

AI and general surgery

Stories of man versus machine, such as the one of John Henry dying trying to better the steam-powered hammer [9], show how machines have long been feared but have eventually come to be welcomed and anxiously expected. By employing techniques that allow for more indirect and complex non-linear relationships and multivariate effects than conventional statistical analysis, ML is particularly useful for identifying subtle patterns in large datasets - patterns that may be imperceptible to humans performing manual analyses [10,11]. By using non-linear models that combine numerous data sources, such as diagnoses, treatments, and laboratory results, ML surpassed logistic regression for predicting surgical site infections (SSI) [12].

Additionally, ensemble ML allows for prediction accuracy levels that are regarded to be impossible with traditional statistics [13]. For instance, to anticipate anastomotic leak following colorectal resections in surgical patients, natural language processing (NLP) has been utilized to automatically trawl through electronic medical records. In clinical trials, artificial neural networks (ANN) beat more traditional risk prediction methodologies dramatically. For example, an ANN's sensitivity (89%) and specificity (96%), for predicting pancreatitis severity six hours after admission, surpassed acute physiology and chronic health evaluation II's (APACHE II) sensitivity (80%) and specificity (85%) [14]. ANNs, in conjunction with other ML techniques, have predicted in-hospital mortality following open abdominal aortic aneurysm (AAA) repair, with a sensitivity of 87%, specificity of 96.1%, and accuracy of 95.4%, utilizing clinical factors, such as patient history, medicines, blood pressure, and duration of stay [15]. While autonomous robotic surgery will likely remain a long way off for some time, cross-disciplinary collaboration will certainly improve AI's potential to complement surgical treatment. The capacity of AI to assess combinations of structured and unstructured data to create clinical decision support accounts for a large portion of its therapeutic potential. To generate innovations, each form of data might be evaluated separately or in conjunction with multiple types of algorithms [16].

The surgical sector has witnessed tremendous and ongoing technical advancements in recent years. The adoption of the Internet of Things (IoT) concept in surgical practice stands out as one of the most revolutionary developments [17]. A subset of IoT identified as the Internet of Surgical Things (IoST) involves the integration of software, sensors, and smart surgical instruments to enhance the effectiveness, safety, and results of surgical procedures. IoST may be beneficial in primary disease diagnosis, particularly in colorectal cancer (CRC). ML could serve as a prompt and effective confirmation test following the initial conventional clinical inquiry while assisting trainee doctors in gaining expertise in CRC diagnosis throughout their internship years. Moreover, global health systems may benefit from the incorporation of DL into CRC evaluation [18]. Further investigation has been conducted into the potential clinical practice implementation of deep learning algorithms for the classification and diagnosis of CRC histopathology images. The advancement made possible by deep learning algorithms has the potential to improve CRC detection's accuracy and efficacy [19].

AI and thoracic surgery

To predict cardio-respiratory morbidity following pulmonary resection for non-small cell lung cancer (NSCLC), Santos-Garcia et al. [20] assessed the effectiveness of an ANN ensemble. They used 348 examples to train their AI and 141 cases to evaluate their model. Prediction sensitivity and specificity were 0.67 (95% CI: 0.49-0.79) and 1.00 (95% CI: 0.97-1.00), respectively. The receiver operating characteristic area was 0.98. Similar to this, Esteva et al. [21] employed four different probabilistic ANN models that were trained using information from 113 NSCLC patients. With the following information, three of the models were programmed to estimate postoperative prognosis (dead/alive): Model 1: 96 clinical and laboratory variables are included; Model 2: using simply the clinical and laboratory variables connected to the risk scores of Goldman et al. [22] and Torrington et al. [23]; and Model 3: utilizing all variables aside from those connected to the risk scores of Goldman et al. [22] and Torrington et al. [23]. To estimate serious postoperative problems, a fourth model, including all variables, was created. All four models were put to the test on 28 patients. Models 1 and 3 properly classified all 28 test instances; however, Model 2 made six classification mistakes out of the 28 test cases. In all 28 test instances, Model 4 properly predicted severe postoperative problems.

In a study by Yu et al. [24], AI software used quantitative histopathology characteristics collected from 2,186 whole-slide pathology pictures from the Cancer Genome Atlas to discriminate between primary lung adenocarcinoma and squamous cell carcinoma. In a multicenter, non-interventional trial, including 120 pulmonologists from 16 hospitals in five different European countries, Topalovic et al. [25] showed that AI performed better than pulmonologists in the interpretation of pulmonary function tests (PFT). Pulmonologists' pattern identification of PFTs met the recommendations in 74.4% of instances, and pulmonologists made proper diagnoses in 44.6% of cases, whereas AI precisely matched the PFT pattern interpretations (100%) and gave a correct diagnosis in 82% of all cases. Due to its precision in identifying tiny patterns, such as in the categorization of lung nodules, ML may be useful in tests relevant to thoracic surgeons [16].

AI and vascular surgery

Imaging is a critical stage in the treatment offered to patients in vascular surgery, helping confirm the diagnosis, evaluate the prognosis, and plan the surgical intervention. AI approaches can assist in enhancing picture segmentation and pattern identification, as well as automate repetitive activities, increasing repeatability and lowering computing time. Several AI-derived algorithms, for example, have been used to enhance aortic aneurysm segmentation, allowing for detailed assessment of aneurysm geometry and morphology [26-30].

ML has also been utilized to create completely automated pipelines for detecting and measuring vascular calcifications in CT-scan pictures [31,32]. Image segmentation and risk classification systems were created for patients with carotid artery stenosis [33-35]. AI has promising applications in picture segmentation, automation, data analysis from medical records, easing and enhancing data gathering, and quantitative measures in huge patient datasets. The danger posed to patients and the results of the operation may be better assessed using a mix of these techniques. Several ML algorithms, for example, have been devised to assess the risk of aortic aneurysm development and rupture or to predict outcomes following aneurysm surgical repair [15,36-40].

A recent study revealed considerable cultural variations in the treatment of juxta-renal AAA, with vascular surgeons recommending either continued monitoring, endovascular or open surgery for the same patient [41]. This highlights the critical need for new technologies to assist surgeons in determining the best treatment strategy. AI may be able to categorize patient states, better estimate the risk of pre- and post-operative problems, and advise surgeons in choosing the most suitable surgical method by enabling the formulation of multi-variable scores incorporating clinical, biological, and imaging parameters. Finally, AI might be used in medical training and teaching by simulating clinical circumstances. Virtual reality simulations, for example, have been developed and might be used to instruct novice surgeons on fundamental endovascular operations [42,43].

AI and urology

AI has been explored and utilized in clinical practice by urology physicians and scientists to help in illness diagnosis and therapy [44,45]. From a diagnostic standpoint, AI can classify prostate tumor histology and lymph node spread using picture segmentation [46]. In a separate use of AI in urologic imaging, De Perrot et al. correctly distinguished phleboliths from kidney stones on low-dose CT scans for patients presenting with acute flank discomfort [47]. AI demonstrated a comparable benefit in diagnosing urinary tract infections [48]. Clinicians use clinical symptoms and urinalysis to guide early therapy rather than waiting 24-48 hours for a urine culture to provide results. AI has been proven to outperform current prediction models for UTI diagnosis, as well as provider judgment when combined with these variables and other patient parameters [49].

AI is being studied as a prediction model for urologic cancer development, as it is in all oncologic domains [50]. AI models tend to be more accurate and comprehensive than standard regression statistics utilized in the evaluation of urologic cancer datasets [51]. In a study of 109 patients, two AI approaches, ANN and neuro-fuzzy modeling (NFM), were compared to established statistical methods for predicting bladder cancer relapse. Both strategies outperformed typical statistical methods in terms of accuracy (88%, 95%, and 71-77%, respectively) [52]. AI is also assisting in the provision of patient-centered care for prostate cancer patients [53].

Early studies in reproductive urology employed AI to find characteristics that predicted sperm quality. Fecundity rates have been shown to be declining over the previous few decades, which served as a major impetus for this program. Gil et al. investigated the use of several AI networks to examine the relationship between environmental variables and/or lifestyle behaviors and their potential impacts on sperm quality [54]. By using an additional optimization approach known as the sperm whale optimization algorithm, Engy et al. have also proven further optimization of an ANN for predicting fertility quality [55].

AI and neurosurgery

Deep learning has lately demonstrated efficacy in a variety of clinical image decision-making models. On CT head examinations, studies have shown that 2D convolutional neural networks (CNN) perform well in detecting intracranial hemorrhage and other acute brain findings, such as mass effect or skull fractures [56-59]. One recent study [60] investigated the possible relevance of deep learning in the identification of cerebral aneurysms using magnetic resonance angiograms.

Robotics and neuronavigation technologies assist in minimally invasive surgery. Neuronavigation, machine vision, and image fusion control the removal of mass lesions and subsequently precision radiotherapy. Machine vision and expert pathology systems provide histological analyses. AI and DM applications check the history against medical records. Expert systems support diagnostic testing [61]. In order to properly assess patients with neurologic injuries, neurocritical care must take into account the complexity of both medical and surgical illness states. Intracranial pressure (ICP), electroencephalograms (EEGs), hemodynamics, ventilation, body temperature, serial neurological assessments, fluid intake-output, and other neurophysiologic parameters are just a few of the numerous data points that the neurocritical care physician may gather using multimodality monitoring (MMM) [61,62].

Radiomics has been proposed to investigate the relationship between medical imaging and underlying genetic features. Radiomics is the term for a method that extracts high-throughput quantitative information from radiographic images and creates prediction models linking image features to genetic patterns and clinical results [63]. A variety of radiomics models have been presented in recent years for survival prediction [64], distant metastasis prediction [65], molecular features classification [66], and so on.

AI and orthopedic surgery

One of the medical specialties with the most cutting-edge technology is orthopedic surgery. Nonetheless, the implementation of AI and ML in orthopedics is still in its early stages [67]. ML may be used to estimate the rate of post-operative complications for each patient, forecast injury risk patterns, assist clinical decision-making, and predict outcomes [68-70]. In several situations, ML not only outperforms orthopedic doctors in fracture identification of the upper limb, ankle, and spine [69] but can also aid in cartilage thickness analysis [71]. Moreover, the remarkable accuracy with which AI models can determine *in situ* implant models can be of immense value in planning revision surgery [72].

Incorporating ML into existing imaging systems makes them "intelligent," enabling quicker imaging rates and the capacity to modify ongoing magnetic resonance imaging sequences to visualize a lesion [73]. This may also be accomplished by including data from a patient's medical records, which allows the software to choose the most appropriate patient-specific imaging examination and methodology [67]. The goal of using ML is to improve workflow and diagnosis accuracy rather than to completely replace radiologists [73]. These algorithms may recognize results that may not be as obvious to the naked eye. For instance, they can predict O6-methylguanine methyltransferase gene promoter methylation in glioblastoma multiforme tumors by analyzing changes in MRI intensity [74]. Two models for categorizing fractures have also been created by several researchers for the purpose of fracture detection [75-77].

AI-controlled robotic systems are commonly utilized in manufacturing lines and biological laboratories [78]. The spread and use of autonomous systems in surgery, on the other hand, has been slower, with most systems, such as the FDA-approved da Vinci surgical system, just translating surgeon hand gestures as a type of robotically aided surgery [79]. Autonomous knot-tying robots have recently been invented, and they are one of the most regularly utilized surgical techniques [78]. With the availability of computer programs such as KeyGene and CellNet, the horizons for AI and ML in regenerative orthopedics are expanding even further [80].

Teaming and computer vision

Computer vision is the ability of computers to develop an understanding of objects and images by recognizing multiple elements in the video or image data and distinguishing them from other visually similar objects [81]. The use of computer vision can replace or complement the already used computer-based imaging technologies, such as MRI, CT, and ultrasound as it is estimated that a single one-minute HD surgical video contains 25 times more data than a CT scan [16].

Computer vision has been shown to provide additional layers of diagnostic power to the doctor, making errors in the detection of abnormalities in anatomical structures less likely. The outcome of the procedures is significantly improved by the augmentation of visual feed from sensors in the computer to the clinician at the human-computer interface, which essentially complements the skills of the pathologists and clinicians. It is estimated that the error rate of detecting abnormalities through lymph nodes has been reduced from 3.4% to 0.5% by the use of AI. A mapping of intricate pathological features can influence the surgeon’s decision for the surgical procedure [16].

The success of an AI program depends upon the data collected through multiple procedures, both in space and time. Visual data collected during procedures in pre-, intra-, and post-operative care can be used to compile complex and rare anatomical abnormalities for aggregation and augmentation for global availability to surgeons [82]. The use of AI systems combined with robotics has been implemented for many surgical procedures, aiding and guiding the surgeon in real time by improving decision-making and providing better precision and safety to the surgeon during the remotely guided procedure.

Role of AI in intraoperative performance and safety

There has been increased research and development on the use of AI in the operating room (OR), especially for linking robotics with AI in cranial, thoracic, maxillofacial, and orthopedic surgeries recently [83-86]. Although it might not be possible for any AI to replace surgeons in complex surgical procedures, it has been shown by Thananjeyan et al. that robots linked with AI can make incisions with extreme precision along with minor suturing [83].

Teleoperation is a comparatively recent application of robotics, AI, and communication technology, especially for purposes where contact between surgeon and patient may be undesirable for community health, such as during the COVID-19 period. The surgeon may operate by receiving real-time sensory information crucial to judgment and their motor operations conveyed to the robots through an interface between the robot and surgeon [16]. Advancements in data transfer and communication through 5G and future generations of communication have strong and reliable potential for use at global distances [16].

In sensitive surgical procedures, augmented reality integrated through an AI can overlay important information such as vitals and hemodynamic state of a patient in real time to the surgeons, which may help them optimize their techniques, as well as decision-making during the surgery. An AI approach applicable in the operation room for highly sensitive prospective intraoperative is the OR black box system, which integrates data from sensors that can collect sounds and images in the room, along with readings from patient monitors that record such things as heart rate and temperature, as well as images inside the patient from the laparoscopy camera, serving to improve safety and efficacy in the OR [87].

Future implications of AI

AII of the abovementioned can be seen as an emerging replacement for human efforts, such as other technologies, especially in the departments of decision-making and reasoning. This particular virtue can revolutionize the use of AI in surgery, essentially taking the role of cognitive skills of a surgeon, and if combined with robotics, as a reliable assistant to the surgeon. Due to certain and obvious safety risks, the unattended use of AI during surgeries may not become a possibility at least in the near future. However, it may take over most of the burden of diagnostic and non-invasive components of surgery, which is theoretically estimated to improve with the widespread implementation of AI.

The exponential growth of data and training of AI demands active use of computers during surgery, which may help evolve AI applications into more reliable and competent tools for surgery, and the improved outcomes over time may prove their reliability and need for involvement in medicine and surgery. The expectations, considering the ethical problems, are that AI linked with robotics and control panels can serve as a vehicle for a more efficacious and safe surgical approach for the surgeon acting as a driver for the whole process, as the technology and its implementation advances.

Role of AI in surgical research

The development of AI programs has numerous clinical as well as educational implementations in teaching hospitals. The ability of AI to catalog and highlight multiple pathologies along their features can help in numerous breakthroughs in surgical research. In addition to decision-making, more focused areas in the uses of computer vision are research applications, such as studying patient cohorts, as well as longitudinal studies by image-based analysis by the ML program [16]. AI has also been implemented in the development of drugs and vaccines, and the development is expected to progress at a much faster rate than normal, especially during the COVID-19 period. AI can be helpful in clinical trials as well, during pharmacotherapeutic research [88]. AI tools can handle patient recruitment and stratification in bulk of data. With evolution through upcoming years, AI can become powerful enough to integrate and analyze patient details to build their 'digital twins' that can be utilized as virtual cohorts for testing drug and treatment safety and efficacy [89].

Challenges

AI and its practical and sustainable application possess some obvious challenges such as financial and maintenance issues that also require training of staff as well as surgeons. It has been found that surgeons are not ready for the imminent integration of AI into their surgical careers. AI requires quality data analysis both at the input level as well as during integration within AI, which can develop bias from both human and machine errors, which over time leads to biased output, perceived as a lack of empathy and improper medical attention toward patients. This raises ethical considerations concerning the corruptible and biased tendencies of AI programs. A clear example of the ethical flaws of AI lies in facial recognition software, which is susceptible to racial discrimination and could be particularly dangerous in plastic surgery [90]. There are also risks such as cyber-attacks on AI framework as well as the evolution of bias in the complex aftermath of the massive digital network that may not be resolvable by simplistic methods [91].

Limitations

Despite the growing hype surrounding AI and its significant integration in healthcare, along with its benefits in patient management, a wider application of AI and its subfields is still hindered by certain shortcomings. For the ideal functioning of this technology in producing appropriate responses and performing certain tasks, a large amount of input data is needed, which includes hospital records and medical reports. Provision of such a substantial amount of personal data to train and create the algorithms may be difficult. In addition, AI algorithms used to train them can result in bias due to the interpretations from the data available and may impact important clinical and surgical decisions [16].

Some algorithms may present their interpretations and conclusions with little or no insight as to why the outcome was predicted, known as the 'black box'. The lack of conclusive evidence linking the variables to the cause-and-effect relationship between them leaves the individuals unable to trust the technology [4]. To overcome such hurdles, surgeons need to attain a sound knowledge of AI and work alongside scientists and AI experts to ensure its best practical use in assessing patient risk factors and making surgical decisions with the utmost precision.

AI is crucially dependent on elements such as the representativeness of the included populations, missing data, and outliers for AI systems to be generalizable across subgroups. Indeed, it is critical to regularly update and feed fresh patient data to algorithms to modify decision-making [92]. To analyze decisions made by machines, surgeons must comprehend how they are made [93]; to achieve this, it is essential to show interest in the subject and work with the technicians, informaticians, and engineers involved.

## Conclusions

AI is rapidly making inroads into various surgical specialties across the world. Emerging trends indicate that using AI technologies can improve patient care by not only strengthening existing pathways but also by driving innovation in surgery. The meteoric rise of AI is marked by its ever-expanding role in the screening, diagnosis, treatment, and prevention of disease - thus potentially improving patient outcomes by reducing morbidity and mortality. Nonetheless, further research is necessary to ensure the optimum and equitable use of AI and to overcome challenges such as data representativeness and generalisability, lack of AI training, human reluctance, automation bias, ethical considerations, and employment uncertainty.
